# Sarcopenia is associated with increased major adverse cardiovascular event incidence in maintenance hemodialysis patients: a prospective cohort study and mediation analysis

**DOI:** 10.3389/fnut.2024.1426855

**Published:** 2024-09-09

**Authors:** Lu Jiang, Zitao Wang, Mengxuan Yuan, Weiping Wang, Buyun Wu, Huijuan Mao

**Affiliations:** ^1^Department of Nephrology, The First Affiliated Hospital of Nanjing Medical University, Jiangsu Province Hospital, Nanjing, China; ^2^Department of Nephrology, Jiangdu People’s Hospital Affiliated to Medical College of Yangzhou University, Yangzhou, China; ^3^Department of Radiology, Jiangdu People’s Hospital Affiliated to Medical College of Yangzhou University, Yangzhou, China

**Keywords:** cohort study, coronary artery calcification score, maintenance hemodialysis, major adverse cardiovascular event, mediation analysis, sarcopenia

## Abstract

**Background:**

Few studies have investigated the relationship between sarcopenia and the incidence of major adverse cardiovascular events (MACE), which are common complications in maintenance hemodialysis (MHD) patients. This study thus explored the association between sarcopenia and MACE in a prospective cohort with mediation analysis.

**Methods:**

Adult MHD patients in Jiangdu People’s Hospital in December 2019 were screened. The exposure was sarcopenia, as defined by the 2019 Asian Working Group. The primary endpoint was the occurrence of MACE, defined as the composite of all-cause mortality or hospital admission with a primary diagnosis of acute myocardial infarction, stroke, or heart failure during a 3-year follow-up period. Multivariate Cox regression analyses were used to test the association between sarcopenia and subsequent MACE incidence. Mediation analyses were used to investigate whether potential mediators influenced the association between sarcopenia and MACE.

**Results:**

Of the 230 patients enrolled, 57% were male, with a median age of 57 years (interquartile range [IQR]: 50 to 66), and a median dialysis vintage of 67 months (IQR: 32 to 119). The prevalence of sarcopenia was 45.2%. The presence of sarcopenia was significantly correlated with age (Spearman’s r = 0.47, *p* < 0.001), C-reactive protein (Spearman’s r = 0.13, *p* = 0.044), serum albumin (Spearman’s r = −0.22, *p* < 0.001), 25(OH) vitamin D (Spearman’s r = −0.26, *p* < 0.001), and coronary artery calcification score (Spearman’s r = 0.20, *p* = 0.002). Over the 3-year follow-up period, MACE were observed in 59/104 (56.7%) patients with sarcopenia and 38/126 (30.2%) patients without sarcopenia (log-rank *p* < 0.001). After accounting for potential confounders, patients with sarcopenia presented a 66% (4–168%, *p* = 0.035) increase in their risk of MACE incidence as compared to non-sarcopenic individuals. However, adjusted mediation analyses did not detect any indication of a causal mediation pathway linking the effects of sarcopenic status on coronary artery calcification score, C-reactive protein, serum albumin, or 25(OH) vitamin D levels to MACE outcomes. Conversely, sarcopenia exhibited a potential direct effect (average direct effect range: −1.52 to −1.37, all *p* < 0.05) on MACE incidence.

**Conclusion:**

These results revealed that the presence of sarcopenia was associated with a higher incidence of MACE in MHD patients. The putative effects of sarcopenia on this cardiovascular endpoint are possibly not mediated by any causal pathways that include vascular calcification, inflammation, hypoalbuminemia, or vitamin D.

## Highlights

What is already known about this subject?

- Sarcopenia was pervalent in patients with end-stage kidney disease receiving maintenance hemodialysis (MHD).
- Patients on MHD were susceptible to major adverse cardiovascular events (MACE).

What does this study add?

- The presence of sarcopenia increases the risk of MACE incidence among MHD patients.
- Sarcopenia, and its three components, was correlated with coronary artery calcification, inflammation, hypoalbuminemia, or vitamin D levels.
- The effects of sarcopenia on MACE are possibly not mediated by coronary artery calcification, inflammation, hypoalbuminemia and vitamin D levels.

How might this impact on clinical practice or future developments?

- Improvement of sarcopenia may decrease MACE incidence in patients with MHD.

## 1 Introduction

End-stage kidney disease (ESKD) is a growing concern in public healthcare, with over 4 million people receiving kidney replacement therapy (KRT) worldwide in 2020, and the use of KRT is forecast to grow to 5.4 million people in 2030 (1, 2). Maintenance hemodialysis (MHD) is a primary KRT modality for patients with ESKD (1). However, the outcomes for these populations have proven unsatisfactory, with numerous complications, particularly with respect to high mortality rates (about 194 per 1,000 person per year in American) associated with the incidence of cardiovascular events (about 233 per 1,000 person per year in American) (1).

Patients with ESKD are also susceptible to sarcopenia (3), a condition attributed to inflammation, metabolic acidosis, protein-energy wasting, and reduced physical activity (4). Sarcopenia manifests as a progressive and generalized skeletal muscle disorder involving accelerated loss of muscle mass, low strength, and low physical performance (5), resulting in a subsequent decline in quality of life. Previous studies have shown that sarcopenia impacts from 1.5 to 68% of MHD patients (3). Sarcopenia in MHD patients is associated with an increased risk of adverse clinical outcomes, including all-cause mortality (3, 6), cardiovascular events (7, 8), rehospitalization (9), and dependency (10). However, the association between sarcopenia and major adverse cardiovascular events (MACE) in MHD has rarely been described (7, 8). Given that cardiovascular disease stands as the leading cause (more than 50%) of mortality in MHD patients (1), it is imperative that this relationship is characterized and that the underlying mechanisms are explored.

Hence, the primary aim of this study was to determine the impact of sarcopenia and its three components on the incidence of MACE in MHD patients. In order to discern the contributory role of sarcopenia in MACE, we also investigated the correlation between sarcopenia and coronary artery calcification score (CACS) values, as this score can serve as an independent predictor of cardiovascular disease (11) and mortality (12, 13). We additionally explored potential causal mediation pathways involving CACS, inflammation markers, malnutrition markers, and vitamin D levels in an effort to better understand the underlying mechanisms linking sarcopenia to MACE incidence.

## 2 Materials and methods

### 2.1 Study population

This prospective cohort study screened clinically stable Chinese outpatients in December 2019 at the blood purification center of Jiangdu People’s Hospital, a tertiary teaching hospital located in southeastern China. We included adult patients with uremia who received regular hemodialysis treatment for a minimum of 6 months, undergoing three sessions per week, each lasting 4 hours. Participants were excluded if they had concurrent malignant tumors, severe infections, severe heart failure (defined as NYHA class >2), or if they were unable to undergo sarcopenia assessment due to limb movement disorders or a history of appendicular bone surgery.

### 2.2 Data collection

Baseline data were collected within 4 weeks after patients completed sarcopenia assessments. Patient demographic information, including sex, age, body mass index, dialysis vintage, primary kidney disease, single-pool urea clearance index (single-pool Kt/V), blood pressure, interdialytic weight gain (IDWG), and comorbid conditions, were collected. Clinical examinations were also conducted encompassing blood routine, liver and kidney function, C-reactive protein (CRP), blood lipids, serum electrolytes, intact parathyroid hormone, iron indices, 25-hydroxy vitamin D [25 (OH) vitamin D], and β2-microglobulin analyses. Nutritional risk screening 2002 (NRS-2002) data were also collected at baseline (14).

CACS measurements were made via 128-slice multidetector spiral CT scanning from the neck to the abdomen, and were assessed based on the Agatston score (15). Calcification was defined as coronary artery CT density ≥ 130 Hounsfield units (Hu) with a calcification area ≥ 1 mm^2^, and scores were assigned according to the CT density value: score 1 (130 Hu – 199 Hu), score 2 (200 Hu – 299 Hu), score 3 (300 Hu – 399 Hu), score 4 (> 400 Hu). CACS was calculated as the product of the calcification area (mm^2^) and the calcification density score. The sum of calcification scores at each slice yielded the total calcification scores.

Sarcopenia was defined as per the 2019 diagnostic criteria established by the Asian Working Group for Sarcopenia (AWGS). Muscle mass was measured using a bioelectrical impedance analysis (BIA) (InbodyS10, InBody Co. Ltd., Seoul, Korea). BIA measurements were obtained at dialysis sessions held in the middle of the week. Patients were told to consume meals 2 h before dialysis to prevent interference from the meal, and they were not permitted to eat while dialysis was being performed. After dialysis, patients were instructed to lie flat on the bed after their post-hemodialysis weight was measured. Thereafter, patients rested for 10 min in the lying position, and then BIA measurements were recorded using an S10 device. Electrodes were attached to both the feet (lateral and medial sides) and the hands (middle finger and thumb). BIA estimates muscle mass indirectly using electrical conductivity. When quantifying muscle mass, appendicular skeletal muscle mass (ASM) was calculated. In addition, ASM was adjusted using height squared (ASM/height^2^). The height-corrected ASM was termed the appendicular skeletal muscle mass index (ASMI).

Muscle strength was measured with an electronic handgrip strength (HGS) meter (Guangdong Xiangshan Weighing Apparatus Group, China). Briefly, patients gripped the meter tightly using the non-fistulation hand (or the dominant hand for the remaining patients). HGS was measured twice before dialysis, and the highest value was used for analysis. HGS was measured by the same operator at the same dialysis session as BIA measurements were taken. Physical performance was measured using a 6-meter walk test, measuring the time required to walk 6 m at the participant’s usual gait speed.

### 2.3 Patient care

These patients received conventional therapies, including the maintenance of adequate hemodialysis, medical treatment, nutritional support, and psychological counseling. Anemia, chronic kidney disease, and mineral and bone metabolism disorders were managed according to established guidelines (16, 17). Hypertension, diabetes mellitus, and dyslipidemia were controlled through appropriate medical interventions. Nutritional support, including oral enteral supplements and intradialytic parenteral nutrition, was provided for patients who were malnourished to ensure they received adequate dietary intake to meet their specific needs. Additionally, psychological counseling was offered to address mental health concerns and improve overall well-being. Patients were encouraged to engage in appropriate physical exercise tailored to their capabilities and health status.

All nutritional, physical, and psychological interventions followed the standard clinical practice protocols at our hospital and were prescribed at the discretion of the specialists involved in their care. These comprehensive care strategies aimed to optimize the overall health and quality of life for patients undergoing hemodialysis, addressing both their physical and psychological needs.

### 2.4 Exposure and endpoint

The exposure was sarcopenia, which was defined as per the AWGS 2019 criteria (18): (1) Reduction in muscle strength: hand grip strength <28 kg for males and < 18 kg for females, which was established through data from 8 Asian cohorts comprising 21,984 participants age ≥ 65 years (19); (2) Decreased physical performance: 6-meter walking speed test speed <1.0 m/s, as this can independently predict rapid cognitive decline (20), and the mean gait speed was 0.96 m/s (significantly higher than 0.8 m/s) in patients with sarcopenia based on the AWGS 2014 criteria (21); (3) Loss of muscle mass: appendicular skeletal muscle mass index (ASMI) < 7.0 kg/m^2^ for males and < 5.7 kg/m^2^ for females, as suggested by the AWGS 2014 criteria (22). Patients with (3) and either (1) and/or (2) can be diagnosed with sarcopenia.

Patients were prospectively followed until December 7, 2022, with the COVID-19 pandemic having been adequately controlled such that none of these patients were infected before this time. The primary endpoint was the incidence of MACE, defined as all-cause death or hospital admission with a primary diagnosis of acute myocardial infarction, stroke, or heart failure (23) within a 3-year follow-up period. The secondary endpoints were all-cause mortality and cardiovascular mortality.

### 2.5 Sample size and statistical analysis

Based on the assumption that MACE occur in 50% of the sarcopenia population and 30% of the non-sarcopenia population over a 3-year follow-up period, with a dropout rate of 5%, a significance level of 0.05 and with 80% power, the required sample size per group was determined to be 221 when the ratio of sample sizes between the sarcopenia and non-sarcopenia groups was 0.75. The 230 patients enrolled in this study thus meet the required sample size criteria.

Categorical variables were presented as numbers and percentages, with comparisons being performed using χ2 tests. Continuous variables were presented as medians (25th, 75th percentile) and compared using the Wilcoxon rank-sum test. Correlations between sarcopenia or its three components and potential predictors of MACE were plotted using a correlation matrix. The cumulative incidence of MACE or all-cause mortality was assessed using the Kaplan–Meier method in patients with and without sarcopenia, and differences between these curves were assessed with the log-rank test. To determine the hazard ratio (HR) for the relationship between sarcopenia and MACE incidence, both univariate and multivariate Cox regression analyses were employed. The utilized multivariate model was adjusted for potential confounders including age, diabetes nephropathy, catheter use, single-pool Kt/V, IDWG, CRP, transferrin saturation, serum albumin, and CACS. Restricted cubic splines with the median as the reference value and four knots at the 5th, 35th, 65th, and 95th were used to flexibly model the associations between MACE incidence and muscle strength, 6-m walking speed, and muscle mass.

To examine whether CACS, CRP, serum albumin, and/or 25 (OH) vitamin D (which were all significantly correlated with sarcopenia and its three components) mediated the association between sarcopenia and MACE, four mediation models were applied (24, 25). The following estimates were described: (1) the average causal mediation effect (ACME), a variable that explains how much of the putative effect of the sarcopenia on MACE is explained by the possible effect of the mediator; and (2) the average direct effect (ADE), a variable that explains how much of the putative effect of the sarcopenia on MACE is still explained by sarcopenia after considering the effect of any given mediator. Four backward stepwise linear regression models were used to establish confounders that were assigned to the mediators, and a backward stepwise Cox regression analysis was employed to identify cofounders that were assigned to MACE. The confounders assigned to the mediators included: age, CRP, IDWG and single-pool Kt/V for the mediator CACS; catheter use, serum albumin, single-pool Kt/V and CACS for the mediator CRP; age, catheter use, single-pool Kt/V, CRP and 25(OH) vitamin D for the mediator serum albumin; diabetes nephropathy, transferrin saturation, and serum albumin for the mediator 25(OH) vitamin D. The confounders assigned to the outcome MACE were CACS, IDWG, diabetes nephropathy, and transferrin saturation, with age and gender as two traditional confounders.

All hypothesis tests were two-tailed, with a significance level set at *p* < 0.05. Statistical analyses were performed using R software, version 4.0.3.

## 3 Results

### 3.1 Baseline characteristics

**Figure 1:**
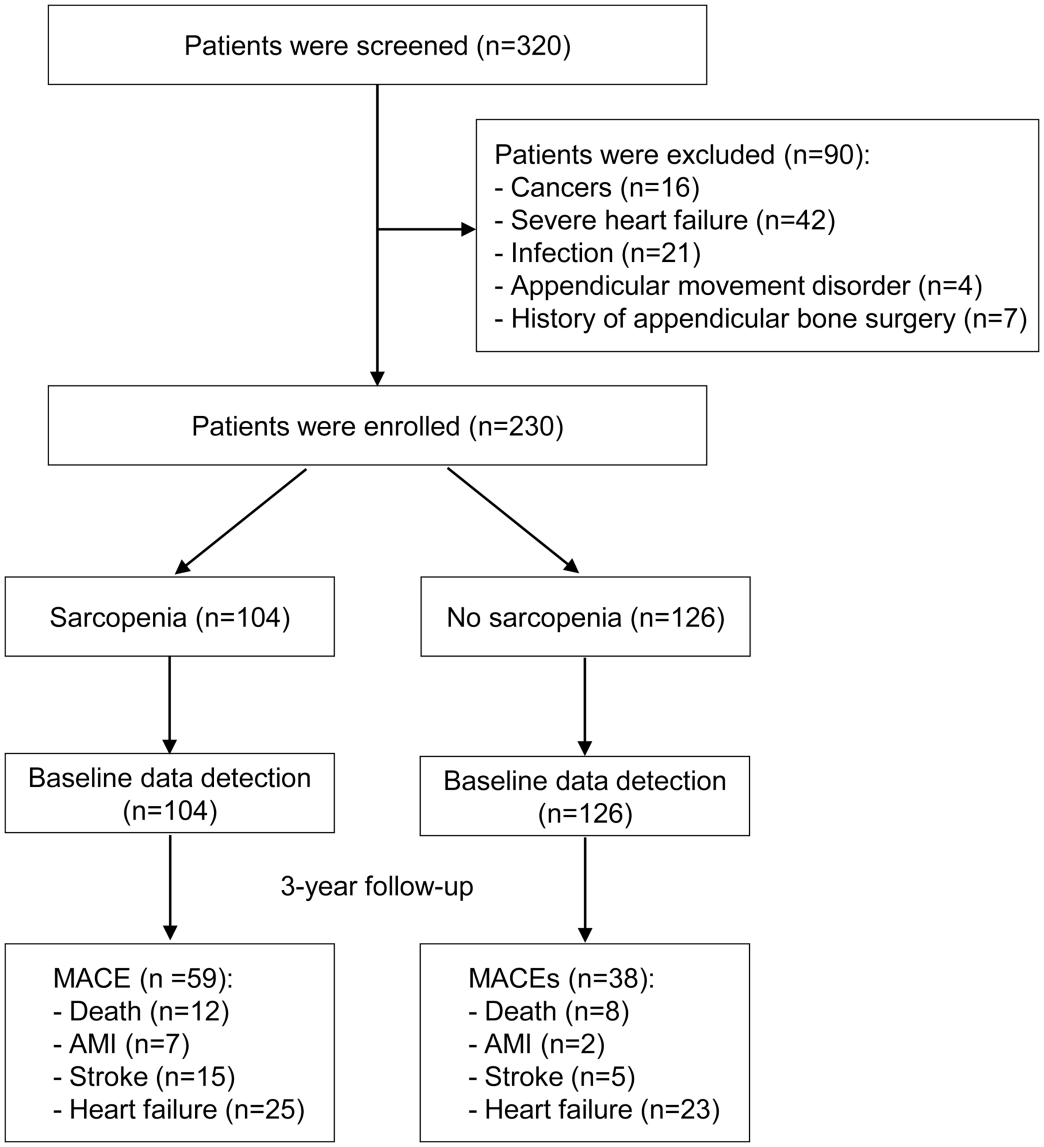
Study flowchart. AMI, acute myocardial infarction; MACE, major adverse cardiac events.

A total of 230 stable MHD patients were ultimately enrolled in this study, including 131 males and 99 females (Figure 1). The median age of the study population was 57 (IQR: 50 to 66) years, with a median dialysis vintage of 67 (IQR: 32 to 119) months. The prevalence of sarcopenia was 45.2%, including 40.4% in males and 51.5% in females. Table 1 exhibited the baseline characteristics of the study patients, grouped according to the presence or absence of sarcopenia. Comparative analysis revealed that patients with sarcopenia exhibited more advanced age, increased catheter usage, elevated CRP levels, reduced albumin levels, decreased 25(OH) vitamin D levels, higher CACS, and higher NRS-2002 scores as compared to non-sarcopenic individuals. Notably, no significant differences were observed between these two groups with respect to the prevalence of diabetes, hypertension, dialysis vintage, body mass index, primary etiology of ESKD, mean blood pressure, IDWG, hemoglobin, serum cholesterol, serum triglycerides, serum electrolytes, intact parathyroid hormone, or serum total carbon dioxide levels (*p* > 0.05) (Table 1).

**Table 1:** Baseline characteristics of MHD patients with and without sarcopenia.

Variables	Non-sarcopenia (*n* = 126)	Sarcopenia (*n* = 104)	*p*-value
**Clinical data**			
Age (years)	52 (47, 59)	65 (57, 72)	**<0.001**
Female (%)	48 (38.1)	51 (49.0)	0.125
Dialysis vintage (months)	70 (32, 119)	65 (33, 116)	0.774
Body mass index (kg/m^2^)	23.3 (19.6, 25.7)	22.8 (20.1, 26.6)	0.705
Causes of kidney failure (%)			0.266
Diabetes nephropathy	16 (12.7)	24 (23.1)	
Glomerulonephritis	97 (77.0)	67 (64.4)	
Hypertension	2 (1.6)	2 (1.9)	
Polycystic Nephropathy	6 (4.8)	7 (6.7)	
Other	5 (4.0)	4 (3.8)	
Hypertension (%)	85 (67.5)	61 (58.7)	0.214
Mean blood pressure (mmHg)	98 (92, 108)	98 (88, 105)	0.090
Current diabetes (%)	15 (11.9)	20 (19.2)	0.175
Catheter use (%)	10 (7.9)	22 (21.2)	**0.007**
Interdialytic weight gain (mL/kg/h)	11.2 (9.8, 12.9)	11.7 (10.1, 13.5)	0.103
Single-pool Kt/V	1.39 (1.21, 1.59)	1.40 (1.24, 1.59)	0.912
Components of sarcopenia			
Appendicular skeletal muscle (kg/m^2^)	8.69 (7.46, 10.33)	5.16 (4.59, 5.79)	**<0.001**
Handgrip strength (kg)	27.3 (21.4, 33.9)	13.2 (7.95, 17.8)	**<0.001**
Gait speed (m/s)	1.08 (0.96, 1.19)	0.86 (0.63, 1.00)	**<0.001**
**Laboratory data**			
Hemoglobin (g/L)	110 (96, 123)	106 (93, 120)	0.201
C-reactive protein (mg/L)	1.51 (0.56, 4.97)	2.73 (0.57, 9.18)	**0.044**
Ferritin (μg/L)	144 (53.6, 429)	181 (54.1, 538)	0.373
Transferrin saturation (%)	32.0 (22.8, 40.9)	31.8 (23.7, 38.3)	0.743
Albumin (g/L)	41.6 (39.6, 44.3)	40.3 (37.6, 42.9)	**0.001**
Blood urea nitrogen (mmol/L)	24.7 (21.0, 29.0)	23.3 (20.1, 29.2)	0.259
Serum creatinine (μmol/L)	930 (763, 1,055)	807 (715, 909)	**<0.001**
Serum urea acid (μmol/L)	435 (385, 481)	429 (369, 481)	0.669
Serum total carbon dioxide (mmol/L)	19.4 (16.4, 21.2)	19.2 (16.7, 21.4)	0.811
Serum potassium (mmol/L)	4.58 (4.11, 5.12)	4.50 (3.94, 4.79)	0.101
Serum calcium (mmol/L)	2.36 (2.23, 2.54)	2.32 (2.23, 2.54)	0.514
Serum phosphorus (mmol/L)	2.11 (1.80, 2.43)	1.97 (1.51, 2.48)	0.061
Calcium-phosphorus product ([mmol/L]^2^)	5.00 (4.17, 5.85)	4.66 (3.28, 5.98)	0.068
Serum total cholesterol (mmol/L)	3.69 (3.08, 4.40)	3.52 (2.95, 4.15)	0.105
Serum triglycerides (mmol/L)	1.94 (1.18, 3.06)	1.72 (1.10, 2.64)	0.202
High-density lipoprotein (mmol/L)	1.06 (0.90, 1.28)	1.02 (0.91, 1.24)	0.448
Low-density lipoprotein (mmol/L)	1.79 (1.43, 2.22)	1.76 (1.43, 2.20)	0.730
25(OH) vitamin D (ng/mL)	28.0 (19.7, 37.3)	21.1 (15.4, 29.7)	**<0.001**
Intact parathyroid hormone (pg/mL)	205 (84.2, 370)	209 (78.8, 338)	0.554
β2-microglobulin (mg/L)	36.0 (28.7, 45.4)	38.5 (32.9, 45.5)	0.068
**Coronary artery calcification score**	33.4 (0.2, 458)	248 (28, 898)	**0.002**
NRS-2002 score	1.0 (1.0, 1.0)	1.0 (1.0, 2.0)	<0.001

Categorical variables are presented as numbers and percentages, while continuous variables are presented as medians (25th, 75th percentile); the bold values refer to *p* < 0.05.

**Figure 2:**
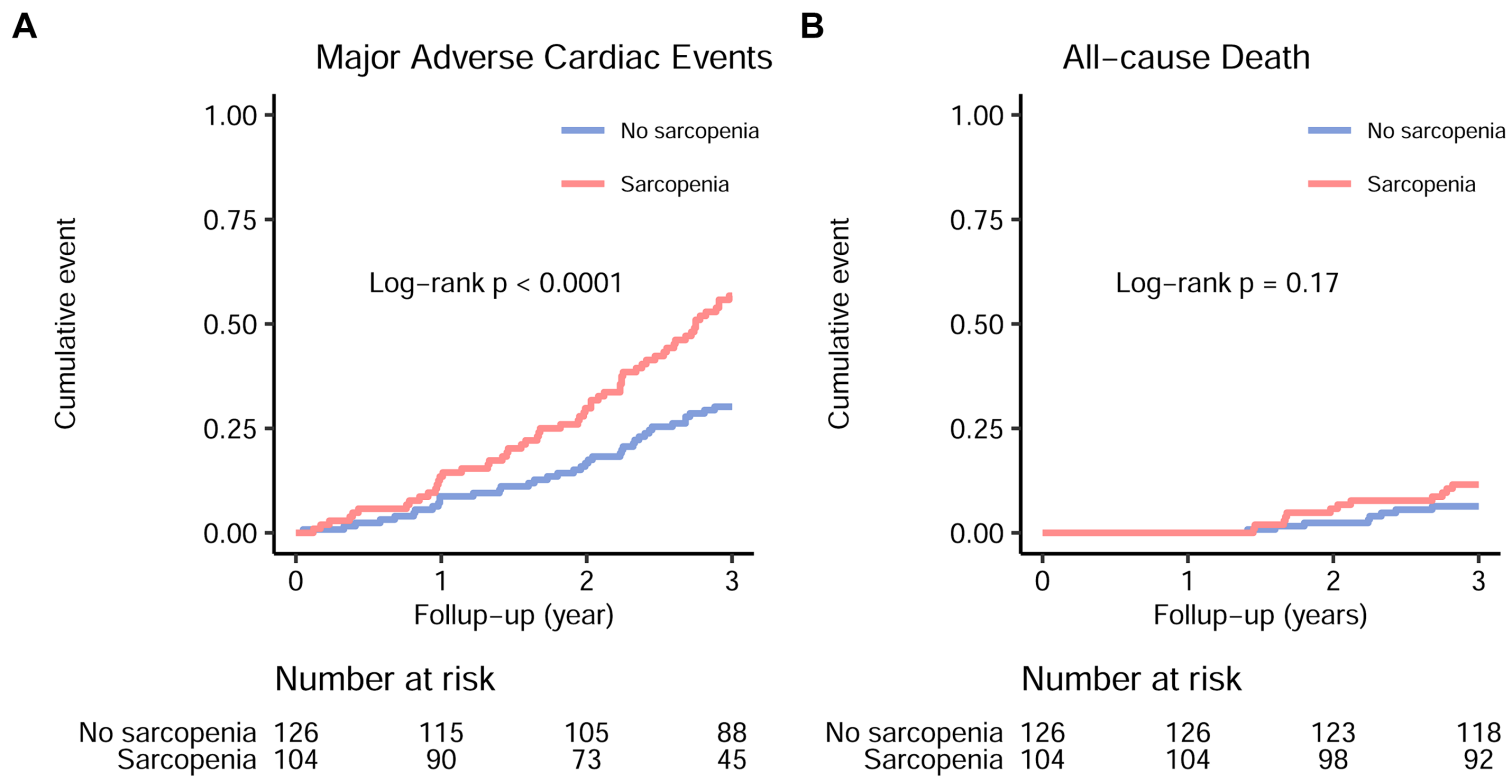
Major adverse cardiac events **(A)** and all-cause mortality **(B)** in maintenance hemodialysis patients with and without sarcopenia. The hazard ratio and 95% confidence interval for major adverse cardiac events and all-cause mortality in sarcopenic individuals were 2.23 (1.48–3.36) and 1.86 (0.76–4.56), respectively.

During the 3-year follow-up period, 59/104 (56.7%) patients with sarcopenia and 38/126 (30.2%) patients without sarcopenia experienced MACE (Figure 2A, log-rank *p* < 0.001). Due to the COVID-19 lockdown measures in China and the fact that our hospital is a regional center, our stable hemodialysis patients had minimal relocation and no loss to follow-up. Supplementary Table S1 displays the differences in baseline characteristics between patients with and without MACE. Individuals who developed MACE exhibited more advanced age, higher rates of diabetic nephropathy as a cause of ESKD, increased catheter usage, lower single-pool Kt/V, higher IDWG, higher CRP levels, reduced transferrin saturation, decreased serum albumin levels, reduced 25(OH) vitamin D levels, higher CACS, and higher NRS-2002 scores. No significant differences were observed between groups with respect to the prevalence of diabetes, hypertension, dialysis vintage, body mass index, mean blood pressure, serum cholesterol, serum triglycerides, serum electrolytes, intact parathyroid hormone, or serum total carbon dioxide (*p* > 0.05).

During the follow-up, 12 out of 104 patients with sarcopenia (11.5%) and 8 out of 126 patients without sarcopenia (6.3%) experienced all-cause mortality (Figure 2B, log-rank *p* = 0.17). Similarly, 11 out of 104 patients with sarcopenia (10.6%) and 8 out of 126 patients without sarcopenia (6.3%) experienced cardiovascular mortality. Due to the small number of events, no further analyses were conducted to examine the association between sarcopenia and all-cause or cardiovascular mortality.

### 3.2 Association between sarcopenia and MACE incidence

As compared with the patients without sarcopenia, those patients with sarcopenia had a higher risk of MACE [unadjusted hazard ratio (HR) = 2.23, 95% CI 1.48–3.36; *p* < 0.001] (Figure 2; Table 2). After adjusting for other predictive variables in a stepwise manner, the risk of MACE incidence remained significantly elevated for sarcopenic patients (adjusted HR = 1.66, 95% CI 1.04–2.68; *p* = 0.036) as compared to non-sarcopenic individuals (Table 2). No significant increase in the risk of all-cause mortality was detected among sarcopenic patients (unadjusted HR = 1.86, 95% CI = 0.76–4.56; *p* = 0.172) (Table 2).

**Table 2:** Hazard ratios for MACE incidence and all-cause death among sarcopenic individuals.

Models	Major adverse cardiac events		All-cause death	
	HR (95% CI)	*p*-value	HR (95% CI)	*p*-value
Model 1	2.23 (1.48–3.36)	<0.001	1.86 (0.76–4.56)	0.172
Model 2	1.82 (1.14–2.89)	0.012		
Model 3	1.72 (1.07–2.76)	0.024		
Model 4	1.66 (1.04–2.68)	0.035		

Model 1: univariate analysis; Model 2: Model 1 with adjustment for clinical factors (including age, diabetes nephropathy, catheter use, sing-pool Kt/V, and interdialytic weight gain); Model 3: Model 2 with adjustment for laboratory indices (including C-reactive protein levels, transferrin saturation, and serum albumin levels); Model 4: Model 3 with adjustment for coronary artery calcification score.

**Figure 3:**
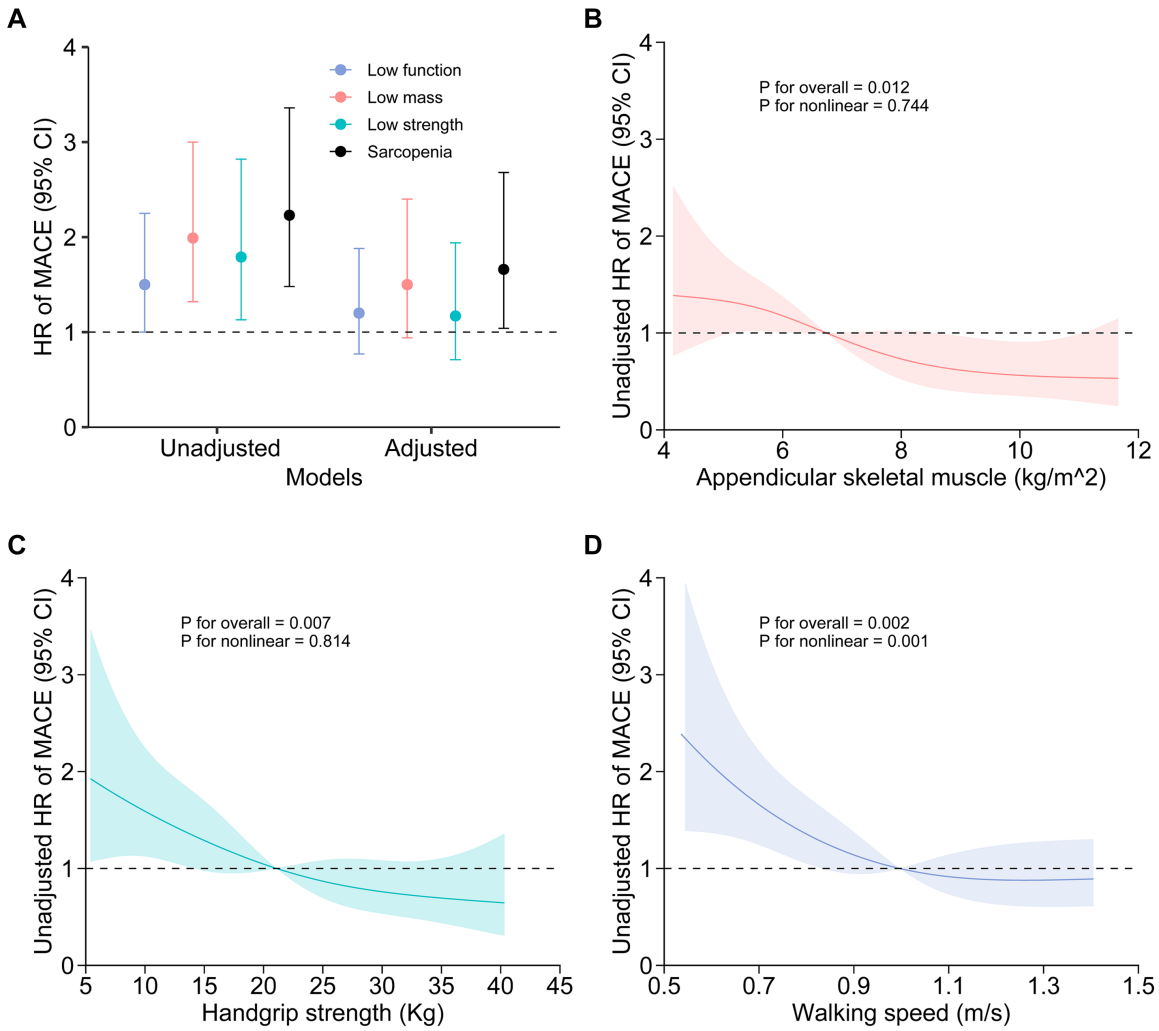
Association between sarcopenia or the components of sarcopenia and MACE incidence. Unadjusted and adjusted hazard ratios for the relationships between sarcopenia components and MACE are presented **(A)**. Age, diabetes nephropathy, catheter use, single-pool Kt/V, interdialytic weight gain, transferrin saturation, serum albumin levels, C-reactive protein levels, and coronary artery calcification scores were accounted for in the adjusted model. Restricted cubic spline visualization revealed a linear relationship between MACE and both appendicular skeletal muscle **(B)** and handgrip strength **(C)**, while also revealing a nonlinear association between MACE and 6-meter walking speed **(D)**.

The three components of sarcopenia, including decreased muscle mass, low muscle strength, and reduced physical function, exhibited significant associations with MACE incidence in univariate Cox models (Figure 3A; Table 3). However, after adjusting for other variables, these associations did not remain significant (Figure 3A; Table 3). Furthermore, a restricted cubic spline analysis revealed a linear relationship between MACE incidence and both appendicular skeletal muscle (Figure 3B) and handgrip strength (Figure 3C), whereas a non-linear association was observed between MACE incidence and 6-meter walking speed (Figure 3D).

**Table 3:** Hazard ratios for MACE incidence and sarcopenia components.

Models	Univariate		Multivariate	
	HR (95% CI)	*p*-value	HR (95% CI)	*p*-value
Low skeletal muscle mass	1.99 (1.32–3.00)	<0.001	1.50 (0.94–2.40)	0.089
Low power grip	1.79 (1.13–2.82)	0.012	1.17 (0.71–1.94)	0.531
Low physical performance	1.50 (1.00–2.25)	0.048	1.20 (0.77–1.88)	0.416

The multivariate model was adjusted for age, diabetes nephropathy, catheter use, single-pool Kt/V, interdialytic weight gain, serum albumin levels, C-reactive protein levels, transferrin saturation, and coronary artery calcification score.

### 3.3 Correlation between sarcopenia and potential predictors of MACE

**Figure 4:**
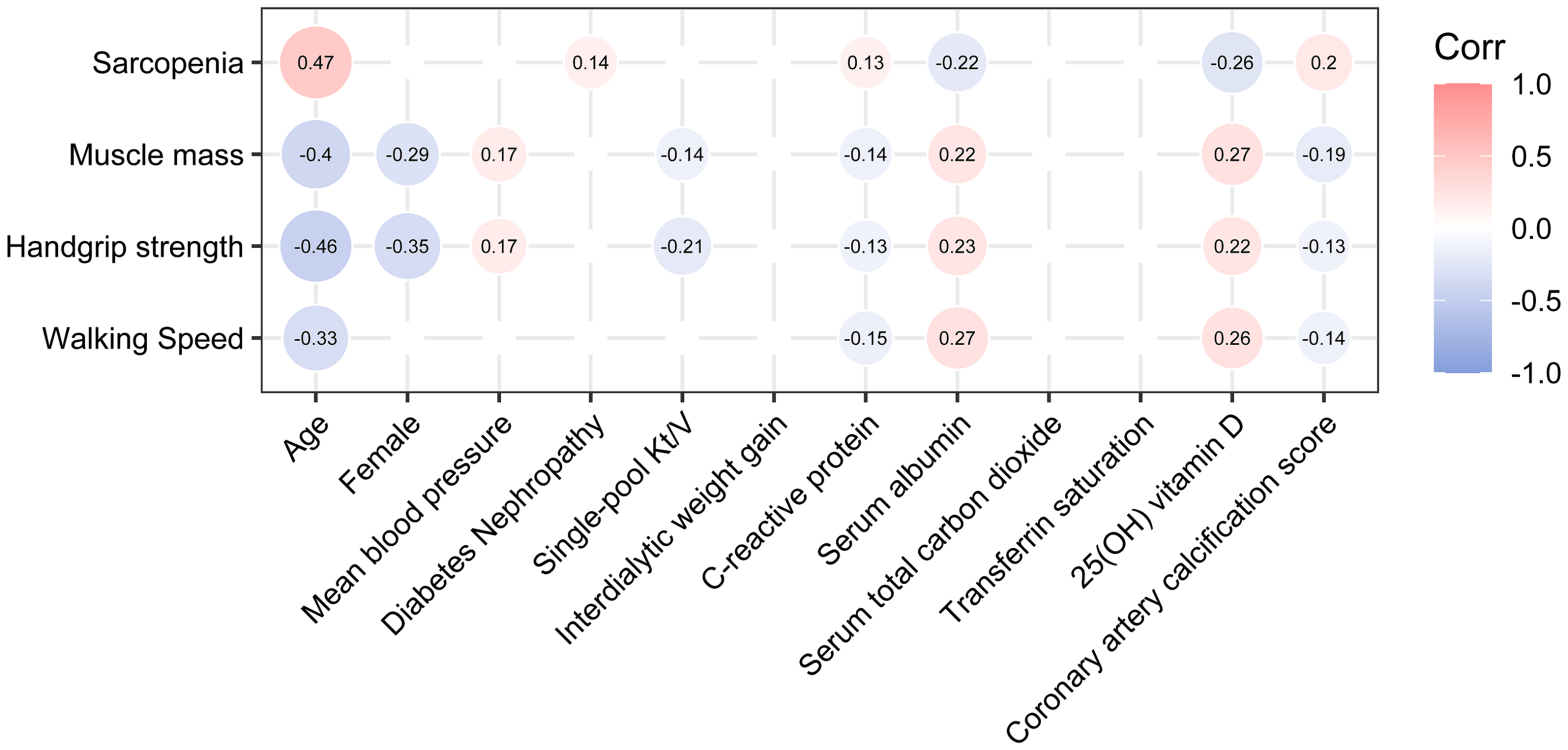
Correlations between sarcopenia or its components and potential predictors of MACE. Only those Spearman’s correlation coefficients with a *p*-value <0.05 are shown. *p-*values were not adjusted, as this was an exploratory analysis.

There was a significant correlation between the presence of sarcopenia and various predictors of MACE, including age (Spearman’s r = 0.47, *p* < 0.001), CRP (Spearman’s r = 0.13, *p* = 0.044), serum albumin (Spearman’s r = −0.22, *p* < 0.001), 25(OH) vitamin D (Spearman’s r = −0.26, *p* < 0.001), and CACS (Spearman’s r = 0.20, *p* = 0.002) in the correlation matrix (Figure 4). All three components of sarcopenia showed associations with these variables. However, no significant correlations were detected between the presence of sarcopenia and gender, mean blood pressure, single-pool Kt/V, IDWG, serum total carbon dioxide, or transferrin saturation.

### 3.4 Mediation analysis

**Figure 5:**
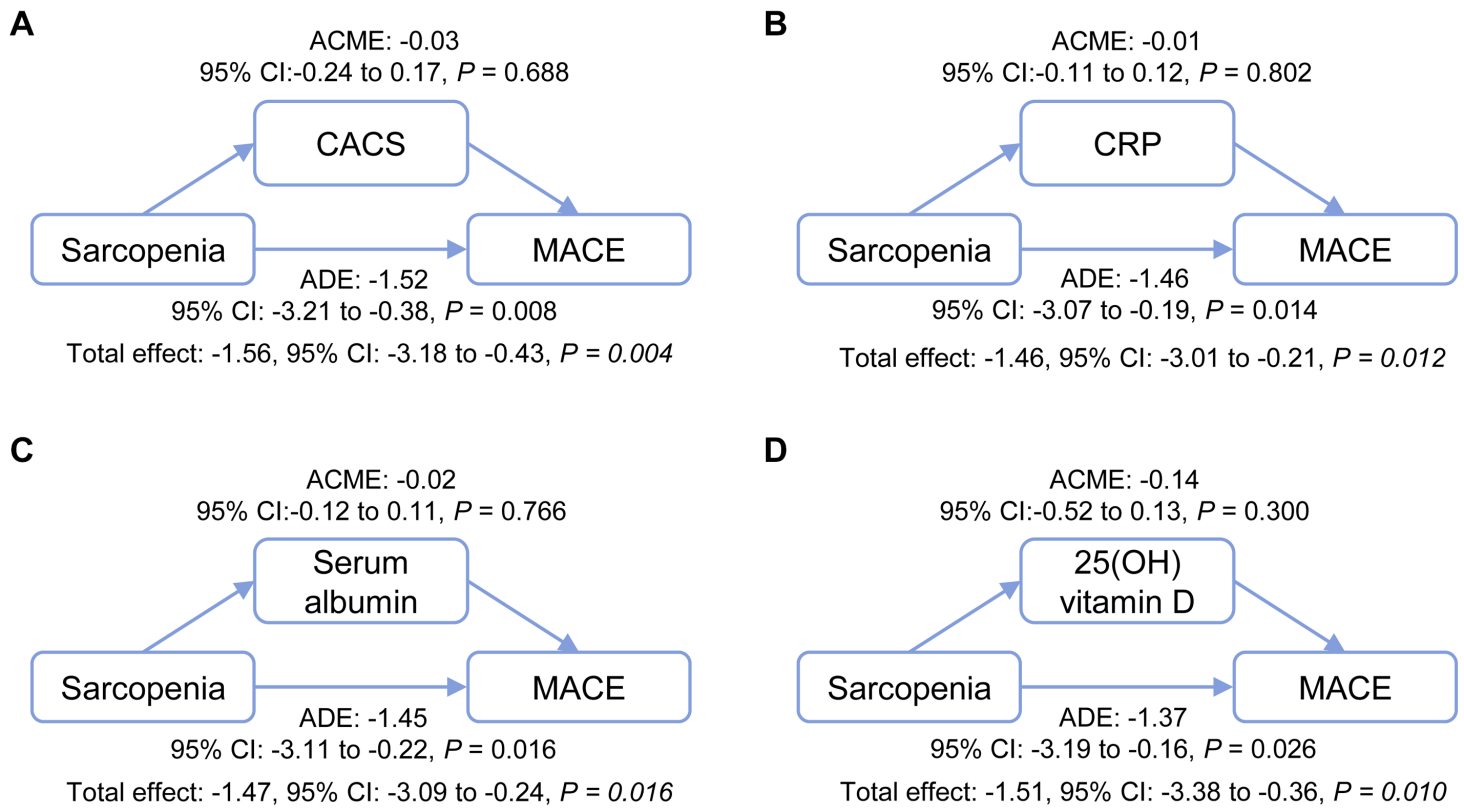
Mediation analysis of the potential mechanisms through which sarcopenia contributes to MACE incidence. The effects of sarcopenia on MACE incidence directly and indirectly through relationships mediated by CACS **(A)**, CRP **(B)**, serum albumin **(C)**, and 25(OH) vitamin D **(D)** were analyzed. ACME, average causal mediation effect; ADE, average direct effect; CACS, coronary artery calcification score; CRP, C-reactive protein; MACE, major adverse cardiovascular events.

To investigate whether the association between sarcopenia and MACE incidence was modulated by the mediating effects of sarcopenia on CACS or other factors, four multivariable mediation models were applied (Figure 5). No causal mediation pathway for this effect was detected through CACS, CRP, serum albumin, or 25(OH) vitamin D (all ACME were not significantly different from zero). In contrast, sarcopenia appeared to have a potential average direct effect (ADE between −1.52 and − 1.37, all *p* < 0.05) on MACE. In all full models, which included 10 confounders—except for the model using 25-OH vitamin D as a mediator (*p* = 0.084)—significant ADEs were observed for the relationship between sarcopenia and MACE, while the ACMEs of the four mediators were not significant (Supplementary Table S2).

## 4 Discussion

Sarcopenia has recently been recognized as a significant public health concern, particularly in hemodialysis patients, with implications for various adverse clinical outcomes (4). However, the association between sarcopenia and MACE incidence in patients on MHD has not been adequately described (7, 8). In this study, we detected a high prevalence of sarcopenia in stable MHD patients and probed its association with subsequent MACE. The putative effects of sarcopenia on MACE were possibly not mediated by a causal pathway involving CACS, CRP, serum albumin, or vitamin D levels. These findings enrich our understanding of sarcopenia in patients with ESKD. Our study indicates that sarcopenia should be considered an important risk factor when using MACE as an endpoint in future research, and that sarcopenia is likely an important target for reducing MACE.

The prevalence of sarcopenia in MHD patients is considerable, ranging from 1.5 to 68% (3). The diagnosis of sarcopenia currently relies on three main factors: loss of muscle mass, muscle weakness, and reduced muscle function. Several working groups and organizations have established evaluation criteria or recommendations for sarcopenia in different regions (18, 26). However, specific criteria for uremic sarcopenia have not been proposed. In line with Chinese ethnic traits, we adopted the 2019 diagnostic criteria recommended by the AWGS, which provided relatively reliable results. Our study demonstrated a high prevalence of sarcopenia among stable MHD patients, with 45.2% overall prevalence, including 40.4% in males, and 51.5% in females. This highlights the significant rates of sarcopenia among stable MHD patients with a long dialysis vintage (median 67 months), which may be attributed to factors such as inflammation, metabolic acidosis, protein-energy wasting, and decreased physical activity (3). These findings emphasize the importance of recognizing and addressing sarcopenia in stable MHD patients.

Previous observational studies have hinted at a connection between sarcopenia and 25-hydroxy vitamin D levels (27, 28). This association was also observed in our cohort, although a recent randomized controlled study indicated that administering 2000 IU/d of vitamin D did not lead to improvements in leg power, strength, or physical performance in 136 low-functioning adults, aged 65–89 years (29). Additionally, our cohort revealed that sarcopenia was linked to advanced age, serum albumin levels, and CRP levels, aligning with earlier studies (9, 30). However, we did not observe any association between sarcopenia and baseline serum total carbon dioxide, which may suggest the absence of metabolic acidosis in MHD patients. In our cohort, we found that sarcopenia and its three components were associated with CACS, an independent risk factor for cardiovascular disease (11) and mortality (12, 13) in MHD patients. This was consistent with previous studies concluding that low skeletal muscle mass was significantly associated with an elevated risk of coronary artery calcification (31, 32) and major adverse cardiac and cerebrovascular events (27).

The association between sarcopenia and MACE incidence in MHD patients has not been thoroughly examined (7, 8). A recent study by Lee et al. demonstrated that serum creatinine/serum cystatin C ratios, a proxy for muscle mass, were associated with cardiovascular and non-cardiovascular death in older patients with coronary artery disease (CAD) (33). A systemic review suggested that sarcopenia was associated with poor MACE outcomes in patients with CAD (34). However, these studies did not adhere to a standardized definition of sarcopenia, limiting the generalizability of their findings. Through a prospective cohort study with a 3-year follow-up, we confirmed that sarcopenia in stable MHD patients was associated with an increased risk of MACE. Furthermore, we observed a linear decrease in MACE rates as muscle mass or strength increased. This suggested that higher muscle mass may be beneficial, regardless of the defined cutoff value for sarcopenia (18, 26). However, external validation of our findings is necessary.

The underlying mechanism by which sarcopenia contributes to MACE incidence remains unclear. Sarcopenia may lead to increased adiposity, insulin resistance, and chronic inflammation, and thus, predispose adults to developing cardiovascular events (35). In patients with ESKD, it has been postulated that inflammation, malnutrition, vitamin D, or vascular calcification may play a role (9, 28, 34). However, our mediation analyses did not support this hypothesis and instead demonstrated a potential direct effect. Moreover, we found that both muscle mass and muscle strength were linearly associated with MACE incidence. One potential explanation for this finding is that reduced skeletal muscle mass leads to diminished endocrine function as a secretory organ that releases myokines and other peptides that may mediate cardiovascular benefits (36). Therefore, individuals with low muscle mass, diminished muscle cell counts, and decreased endocrine function may face a higher risk of MACE. This hypothesis warrants confirmation in future studies.

There are several limitations to our study. Firstly, this was a single-center study, and the sample size was not sufficiently large, necessitating external validation of the results. Secondly, causality cannot be established, even with the prospective cohort design of our study and comprehensive baseline data, and confounding factors were not entirely adjusted for. Thirdly, none of the included biomarkers explained the pathogenesis of MACE in these patients, limiting our ability to interpret the relationship between sarcopenia and MACE. Fourth, repeated assessments of sarcopenia and other nutrition-related markers were not conducted to provide a complete picture of patient health status and the progression of sarcopenia. Lastly, the influence of other unconsidered mediating variables cannot be entirely ruled out by only including four mediators in our mediation analysis. Our study does not imply that other potential mediating variables do not exist, nor does it suggest that all effects of sarcopenia on MACE are direct. Therefore, we cannot conclusively state a direct relationship between sarcopenia and MACE. This relationship necessitates further confirmation through interventional studies, such as prospective trials incorporating promising exercise therapies or drugs for sarcopenia, in order to determine whether they can mitigate the risk of MACE in MHD patients.

## 5 Conclusion

Our prospective cohort study demonstrated that the presence of sarcopenia increases the risk of MACE incidence among stable MHD patients. Sarcopenia, and its three components, was associated with coronary artery calcification, inflammation, hypoalbuminemia, or vitamin D levels; but its effect on MACE is possibly not mediated by the above four pathways. Improvement of sarcopenia may decrease MACE incidence in patients with MHD.

## Data Availability

The raw data supporting the conclusions of this article will be made available by the authors, without undue reservation.
