# Supplementary material for: Sarcopenia is associated with increased major adverse cardiovascular event incidence in maintenance hemodialysis patients: a prospective cohort study and mediation analysis
Source: Front Nutr. 2024 Sep 9;11:1426855. doi: 10.3389/fnut.2024.1426855 (PMC11417030; doi:10.3389/fnut.2024.1426855)
Supplement: Supplementary file 1 [file Table_1.docx]

**Sarcopenia is associated with increased major adverse cardiovascular event incidence in maintenance hemodialysis patients: A prospective cohort study and mediation analysis**

Lu Jiang, Zitao Wang, Mengxuan Yuan, Weiping Wang, Buyun Wu, Huijuan Mao

**Supplementary Materials**

**Supplementary Table S1** Baseline characteristics of MHD patients with and without MACE.

| Variables | No MACE (n=133) | MACE (n=97) | p-value |
| --- | --- | --- | --- |
| **Clinical data** |  |  |  |
| Age (years) | 55 (49, 62) | 59 (52, 71) | **0.002** |
| Female gender (%) | 57 (42.9) | 42 (43.3) | 1.000 |
| Dialysis vintage (months) | 68 (34, 126) | 65 (31, 111) | 0.416 |
| Body mass index (kg/m^2^) | 23.4 (20.0, 26.4) | 22.7 (19.7, 25.9) | 0.306 |
| Causes of kidney failure (%) |  |  | **0.036** |
| Diabetes nephropathy | 16 (12.0) | 24 (24.7) |  |
| Glomerulonephritis | 105 (78.9) | 59 (60.8) |  |
| Hypertension | 1 (0.8) | 3 (3.1) |  |
| Polycystic Nephropathy | 7 (5.3) | 6 (6.2) |  |
| Other | 4 (3.0) | 5 (5.2) |  |
| Hypertension (%) | 85 (63.9) | 61 (62.9) | 0.984 |
| Mean blood pressure (mmHg) | 97.3 (90.7, 105) | 101 (89.7, 113) | 0.145 |
| Diabetes (%) | 16 (12.0) | 19 (19.6) | 0.165 |
| Catheter use (%) | 12 (9.0) | 20 (20.6) | **0.021** |
| Interdialytic weight gain (mL/kg/h) | 11.1 (9.5, 12.8) | 11.8 (10.2, 13.6) | **0.031** |
| Single-pool Kt/V | 1.44 (1.26, 1.62) | 1.35 (1.18, 1.50) | **0.008** |
| Sarcopenia (%) | 45 (33.8) | 59 (60.8) | **<0.001** |
| Components of sarcopenia |  |  |  |
| Appendicular skeletal muscle (kg/m^2^) | 7.45 (5.57, 9.81) | 6.01 (5.01, 7.80) | 0.001 |
| Handgrip strength (kg) | 23.5 (14.9, 31.0) | 18.1 (10.5, 25.3) | <0.001 |
| Gait speed (m/s) | 1.01 (0.90, 1.15) | 0.95 (0.71, 1.11) | 0.009 |
| **Laboratory data** |  |  |  |
| Hemoglobin (g/L) | 110 (97, 123) | 106 (93, 118) | 0.068 |
| C-reactive protein (mg/L) | 1.05 (0.50, 3.68) | 5.03 (1.08, 11.4) | **<0.001** |
| Ferritin (μg/L) | 158 (51.9, 516) | 152 (55.7, 438) | 0.792 |
| Transferrin saturation (%) | 33.1 (26.4, 42.0) | 28.7 (20.2, 36.7) | **0.003** |
| Albumin (g/L) | 41.5 (39.3, 44.1) | 40.5 (37.9, 42.9) | **0.008** |
| Blood urea nitrogen (mmol/L) | 24.4 (20.7, 28.2) | 24.4 (20.3, 29.4) | 0.734 |
| Serum creatinine (μmol/L) | 909 (780, 1038) | 806 (659, 948) | **0.001** |
| Serum urea acid (μmol/L) | 427 (389, 471) | 436 (366, 492) | 0.535 |
| Serum potassium (mmol/L) | 4.48 (4.00, 4.93) | 4.59 (4.17, 5.10) | 0.387 |
| Serum calcium (mmol/L) | 2.39 (2.24, 2.55) | 2.31 (2.19, 2.50) | 0.114 |
| Serum phosphorus (mmol/L) | 2.04 (1.69, 2.38) | 2.02 (1.64, 2.54) | 0.810 |
| Calcium-phosphorus product ([mmol/L]^2^) | 4.92 (4.00, 5.80) | 4.70 (3.56, 6.04) | 0.856 |
| Serum total cholesterol (mmol/L) | 3.63 (3.00, 4.33) | 3.68 (3.01, 4.20) | 0.962 |
| Serum triglycerides (mmol/L) | 1.93 (1.13, 2.93) | 1.77 (1.16, 2.66) | 0.657 |
| High-density lipoprotein (mmol/L) | 1.10 (0.90, 1.31) | 1.01 (0.90, 1.19) | 0.150 |
| Low-density lipoprotein (mmol/L) | 1.74 (1.43, 2.16) | 1.78 (1.43, 2.24) | 0.698 |
| 25(OH)vitamin D (ng/ml) | 27.1 (19.1, 37.2) | 20.9 (15.4, 30.3) | **0.001** |
| Intact parathyroid hormone (pg/mL) | 220 (86, 346) | 203 (76, 349) | 0.923 |
| β2-microglobulin (mg/L) | 36.2 (29.5, 44.9) | 40.3 (32.3, 47.3) | 0.134 |
| **Coronary artery calcification score** | 35 (0, 312) | 508 (28, 2068) | **<0.001** |
| NRS-2002 score | 1.0 (1.0, 1.0) | 1.0 (1.0, 2.0) | **<0.001** |

The categorical variables were presented as numbers and percentages, and continuous variables were presented as medians (25th, 75th percentile); the bold values refer to P<0.05.

**Supplementary Table 2**. Mediation analysis of the potential mechanisms through which sarcopenia contributes to MACE incidence using a three-step stepwise approach.

| Models | **ACME** | **P-value** | **Prop. Mediated** | **ADE** | **P-value** |
| --- | --- | --- | --- | --- | --- |
| Mediator: CACS | | | | | |
| Model 1 | -0.04(-0.28,0.19) | 0.744 |  | -1.67(-3.42,-0.52) | 0.004 |
| Model 2 | -0.03(-0.24,0.17) | 0.688 |  | -1.52(-3.21,-0.38) | 0.008 |
| Model 3 | -0.03(-0.24,0.14) | 0.690 |  | -1.29(-3.26,-0.09) | 0.030 |
| Mediator: CRP | | | | | |
| Model 1 | -0.01(-0.16,0.14) | 0.810 |  | -1.72(-3.56,-0.36) | 0.006 |
| Model 2 | -0.01(-0.11.0.12) | 0.802 |  | -1.46(-3.07,-0.19) | 0.014 |
| Model 3 | -0.01(-0.13,0.11) | 0.840 |  | -1.30(-3.17,-0.13) | 0.030 |
| Mediator: Serum albumin | | | | | |
| Model 1 | -0.04(-0.20,0.11) | 0.600 |  | -1.76(-3.50,-0.48) | <0.001 |
| Model 2 | -0.02(-0.12,0.11) | 0.766 |  | -1.45(-3.12,-0.22) | 0.016 |
| Model 3 | -0.01(-0.09,0.10) | 0.976 |  | -1.30(-2.92,-0.10) | 0.032 |
| Mediator: 25-OH vitamin D | | | | | |
| Model 1 | -0.25(-0.74,0.00) | 0.038 | 0.14(0.00,0.54) | -1.56(-3.21,-0.28) | 0.024 |
| Model 2 | -0.14(-0.52,0.13) | 0.300 |  | -1.37(-3.19,-0.16) | 0.026 |
| Model 3 | -0.12(-0.51,0.15) | 0.400 |  | -1.16(-3.19,0.20) | 0.084 |

Model 1: accounting for age and gender. Model 2: model 1 additionally accounting for cofounders CACS, IDWG, diabetes nephropathy, and transferrin saturation which were obtained using backward stepwise Cox regression analysis. Model 3: model 2 additionally accounting for catheter use, single-pool Kt/V, serum albumin and C-reactive protein levels.
